# Supplementary material for: Modeling and simulation of neocortical micro- and mesocircuitry (Part II, Physiology and experimentation)
Source: eLife. 2026 Jan 20;13:RP99693. doi: 10.7554/eLife.99693 (PMC12818871; doi:10.7554/eLife.99693)
Supplement: Supplementary file 1. [file elife-99693-supp1.pdf]

Table of predictions made by the study.

**(1)**	
Prediction	**Correlation between excitatory and inhibitory populations increases exponentially from supra to infragranular layers; the power of low frequency oscillations also increases towards deeper layers.**
Importance	Charazterizing the correlation between excitatory and inhibitory populations is critical for our understanding of the neural code and is of high interest to theoretical point neuron modellers and experimentalists (***Vogels and Abbott, 2009***; ***Vogels et al., 2011***). Particularly, such correlations are thought to govern homeostasis ("E/I balance") and in turn the dynamics of populations during resting and evoked activity. Moreover, such correlations can give clues towards the level of recurrent processing within a neural population. Despite its perceived importance in the field, a laminar characterization of such correlations over a full spectrum of temporal frequencies is beyond existing techniques for spiking activity. Spike sorting usually only finds a small handful of neighbouring E and I neurons within each cortical layer, which is insufficient to quantify local intra-layer inter-population correlations. Calcium imaging does not have the required temporal resolution for such a characterization and is limited at deeper layers. Unfortunately, only proxies of such correlated neuronal activity can currently be studied, although this still has high impact appeal, due to the described relevance of this question. For example, a recent study analyzed the local field potential (LFP) to demonstrate "a ubiquitous spectrolaminar motif of local field potential power across the primate cortex" (***Mendoza-Halliday et al., 2024***). Particularly, the authors found a decrease in the frequency of the dominant LFP power, from supra- to infra-granular layers. Although this piece of evidence was demonstrated only for noise in the extracellular space (LFP), which was generated by neural activity, this piece of evidence excitingly supports the more detailed prediction of our model where we observed an increase in low frequency oscillations towards deeper layers. The presence of such a hetero-laminar effect also demonstrates the utility of our model: point neuron modellers can now explore the role of different neruon types and connectivity motifs in excitatory/inhibitory balance within a calibrated and detailed model.
Novelty	Our model allows access to the spiking activity of all neurons across layers, for a well calibrated activity state. The detail of our model allow us to explore the correlation and interaction between different morphoelectric neuron types with full spatiotemporal resolution.
Experiment	Beyond the recent LFP study, the quantitative predictions which our model makes will be able to be tested once recording techniques provide suffient clarrity of neuronal population activity. For example, the rapid improvement of extracellular recording devices and spike sorting techniques will hopefully make it possible to soon measure sufficient numbers of E and I neurons within each layer.
Extension	1. Fit exponentials to the data in Figure 4I to predict the precise layer dependence. 2. Extend to look at the cortical depth dependence of spiking correlations.

**(2)**	
Prediction	**Specific spatial targeting rules observed for different inhibitory subpopulations in electron microscopy act to more strongly self-inhibit inhibitory populations and inhibit excitatory populations. This changes E/I balance for inhibitory populations from an excitation dominated state to an inhibition dominated state, whilst overall excitatory populations became less dominated by excitation. Specifically, increased perisomatic targeting by PV+ neurons, increased VIP+ targeting of other inhibitory neurons, neurons in L1 connecting more monosynaptically, and I connections onto E neurons being sparser and closer to the soma. This increased self-inhibition towards central layers with the greatest effect in L4. As a result, inhibitory populations require increased non-local drive.**
Importance	Highly specific targeting rules and self-inhibition by inhibitory populations are likely to play important roles in cortical function. However, it is currently challenging to manipulate the connectivity of inhibitory subpopulations as a way of probing their causal effect. The fact that the firing rates of inhibitory populations increased upon connection of the network in the original connectome but decreased upon connection in the SM-connectome shows that the more specific targeting rules act to make inhibitory populations more inhibited overall by local connectivity, rather than excited.
Novelty	To our knowledge this is the first comparison of two models where one includes spatially specific targeting rules and one that does not. The new calibration technique allowed methodical comparison of the two networks over a 60 different regimes in which the firing rates of the two networks were matched.

(3)

Prediction — **Quantitative prediction of extracellular recording bias.**

Importance — It has commonly been discussed that extracellular recording and spike sorting are likely to bias our view of in vivo spiking activity (***Wohrer et al., 2013***). This has serious consequences for our interpretation of sorted spike trains, which we plan to investigate further.

Novelty — Whilst synthetic datasets have been used to benchmark spike sorting algorithms before, this model is the first to capture the variety of M-types and E-types, combined with realistic in vivo dynamics.

Experiment — This would require large-scale unbiased patch-clamping combined with extracellular recording and spike sorting.

(4)

Prediction — **The degree of participation in high-dimensional simplices predicts the level of correlation between inhibitory and excitatory populations in a cortical layer.**

Importance — Internal “recurrent” processing is of great interest to theoretical modellers because such recurrent processing is thought to underlie many cognitive functions. To study the factors underlying such recurrent processing, theoretical modellers commonly use networks of abstract point neurons with random or all-to-all connectivity. It is important to study how three-dimensional biological design influences the spatiotemproal dynamics of recurrent processing to better understand complex cortical function. Our prediction suggests that pairwise connectivity statistics are insufficient to predict the level of correlation within a cortical layer, and that specific high-dimensional local connectivity motifs drive internal spontaneous activity.

Novelty — Such an analysis is currently unattainable *in vivo* because: 1. consistent electron micropscopy datasets across cortical layers are not available, and 2. the spiking activity over large intra-layer populations is limited (as described for the previous prediction). To our knowledge our analysis represents the most biophysically-constrained *in silico* comparison between high-dimensional connectivity motifs and emergent activity.

Experiment — Larger electron-microscopy datasets which are currently being collected will enable direct comparisons with the dimensionality of the connectivity motifs within a cortical layer. Quantifying E/I spiking correlations at the population level will require better tools for measuring *in vivo* spiking activity, as described for the previous prediction.

(5)

Prediction — **Higher order structure has functional implications**

Importance — The higher order network metrics that have been found relevant in describing the non-random complexity of the network and detailed in the accompanying manuscript have been linked to function. The importance is two-fold: First, it gives relevance to the many ways in which the structure of connectivity has been characterized as non-random before. We now know that this is no mere epiphenomenon, but a mechanism of cortical processing. Second, it provides useful metrics for the description of how structure shapes function. More specific predictions of the link between higher-order network structure and function:

- E/I correlations can be predicted from higher order centrality metrics.
- The strength of a connection can be predicted from how it is embedded in the whole network in a higher order sense. This finding has been further verified in ***MICrONS-Consortium et al.*** (***2021***) (see ***Ecker et al.*** (***2024***))
- Higher order interactions shape the correlation of activity. This has also been verified in ***MICrONS-Consortium et al.*** (***2021***) (see ***Egas Santander et al.*** (***2024***)).

Novelty — In all these structure-function predictions highlighted, the use of higher-order structures was crucial.

(6)

Prediction — **Layer-wise populations receive external drive from non-local connectivity within a precise and specific range. Moreover, input from non-local connectivity is essential for stable non-zero activity at resting.**

Importance — To our knowledge, the amount of conductance that a neuron receives from non-local vs local connectivity is unknown, as it is unknown how a cortical brain region would behave when disconnected from other brain regions. These two factors are important for the constraint of biophysically-detailed models, and the insights they can make. Although it seems trivial that a cortical area should be silent under *in vivo* conditions when disconnected from other areas, we have received questions from experimentalists about whether this is the case, and to our knowledge it is unknown.

Novelty — This was only possible to test with a model at the level of an entire cortical subregion, which incorporated both local and non-local connectivity.

Experiment — This could be estimated *in vivo* by measuring the frequency of synaptic activations across dendrites during resting state activity. To test the need for afferent input from non-local connections for non-zero resting state activity, one would need to ablate incoming connections to a cortical region and measure spiking activity.

(7)

Prediction — **Cortical curvature does not predict level of correlative activity in a cortical column (negative result not shown).**

Importance — The cortex is a highly and heterogeneously curved structure, especially in humans. To our knowledge there have been no studies of the effect of such curvature on spiking activity, for example in terms of the level of correlated activity. If such an effect exists in rodent, it is likely to play out even stronger in human. Even in our model of a single cortical region we observed high heterogeneity in curvature, which we quantified in the first manuscript. However, we found that activity metrics were remarkably unaffected by the resulting differences in connectivity.

Novelty — We are not aware that this potential aspect of cortical function has been considered before.

Experiment — As with the previous point, this could be confirmed or rejected by specifically generating co-registered connectome and activity dataset for regions with different curvature.

(8)

Prediction — **Single whisker deflections activate 5-10% of thalamic fibres in a single barreloid. Excitatory populations respond more sparsely to whisker deflections than inhibitory populations. Stimulus response sparsity is heterogeneous across layers.**

Importance — These predictions are about unknowns of cortical processing within the canonical barrel system.

Novelty — This highlights that critical details about basic cortical processing remain unknown but can be predicted by well constrained cortical models.

Experiment — Testing these predictions would require calcium imaging over VPM barreloids and different layers of cortex.

(9)

Prediction — **L4 PCs contribute to L23 PC response through direct excitatory connections rather than disinhibition. L2/3 PCs and L4 PCs make similar contributions to L2/3 PC responses, L5 PCs have a smaller effect, L6 PCs' have a negligible effect.**

Importance — This relates to the mechanisms underlying canonical cortical processing.

Novelty — This demonstrates the model's capacity for experiments that are currently limited by existing *in vivo* techniques as described in the manuscript.

Experiment — These predictions are grounded in techniques which could become available in the near future (markers for the optogenetic tagging of different cell types).

(10)

Prediction — **Encoding of synchronous and rate-code signals by inhibitory subpopulations is strongest for PV+, followed by 5HT3aR+, and then SSt+.**

Importance — Encoding by different inhibitory neuron-types is likely to play a key role in how the cortex processes and represents information *in vivo*.

Novelty — The study which inspired this work could only be performed *in vitro*. That is, to our knowledge, stimulating precise multi-neuron patterns of spikes and measuring spiking responses from different inhibitory sub-types would be challenging to perform *in vivo*.

Experiment — Voltage-imaging may soon make such a study simpler to perform *in vivo*.

**(11)**	
Prediction	**Quantitative predictions about the nature and connectivity underlying correlated activity.** Particularly, we predict 1) that correlations between afferent regions, increases internal correlations, 2) local correlation is predicted by participation in (or vicinity to) a rich club of cortical columns connected by dense non-local connectivity, 3) the distance dependence of spiking correlations.
Importance	The importance, novelty and means of testing predictions about correlated activity have been discussed in other points.

**(12)**	
Prediction	**Specific targeting rules by inhibitory subpopulations increase stimulus-evoked responses in L23, decrease responses in L5 and L6, and slightly increase stimulus response latencies.**
Importance	As discussed for prediction (2) the role of specific targeting rules are important to understand but difficult to study *in vivo*.

References

Ecker A, Egas Santander D, Bolaños-Puchet S, Isbister JB, Reimann MW. Cortical cell assemblies and their underlying connectivity: An in silico study. PLOS Computational Biology. 2024; 20(3):e1011891.

Egas Santander D, Pokorny C, Ecker A, Lazovskis J, Santoro M, Smith JP, Hess K, Levi R, Reimann MW. Heterogeneous and higher-order cortical connectivity undergirds efficient, robust and reliable neural codes. bioRxiv. 2024; doi: 10.1101/2024.03.15.585196.

Mendoza-Halliday D, Major AJ, Lee N, Lichtenfeld MJ, Carlson B, Mitchell B, Meng PD, Xiong Y, Westerberg JA, Jia X, et al. A ubiquitous spectrolaminar motif of local field potential power across the primate cortex. Nature Neuroscience. 2024; p. 1–14.

MICrONS-Consortium, Bae JA, Baptiste M, Bishop CA, Bodor AL, Brittain D, Buchanan J, Bumbarger DJ, Castro MA, Celii B, et al. Functional connectomics spanning multiple areas of mouse visual cortex. bioRxiv. 2021; p. 2021–07.

Vogels TP, Abbott L. Gating multiple signals through detailed balance of excitation and inhibition in spiking networks. Nature neuroscience. 2009; 12(4):483–491.

Vogels TP, Sprekeler H, Zenke F, Clopath C, Gerstner W. Inhibitory plasticity balances excitation and inhibition in sensory pathways and memory networks. Science. 2011; 334(6062):1569–1573.

Wohrer A, Humphries MD, Machens CK. Population-wide distributions of neural activity during perceptual decision-making. Progress in neurobiology. 2013; 103:156–193.
