# Supplementary material for: Modeling and simulation of neocortical micro- and mesocircuitry (Part II, Physiology and experimentation)
Source: eLife. 2026 Jan 20;13:RP99693. doi: 10.7554/eLife.99693 (PMC12818871; doi:10.7554/eLife.99693)
Supplement: Supplementary file 2. — Average class parameters are marked in bold and are used predictively (in lack of reference in vitro data) for the remaining pathways belonging to the same class. Physical dimensions are as follows: peak conductance g^\begin{document}$\hat{g}$\end{document}: nS, depression and facilitation time constants D\begin{document}$D$\end{document}, F\begin{document}$F$\end{document}, and the EPSC τdecay\begin{document}$\tau_{decay}$\end{document}: ms, the Hill coefficient of the nonlinear [Ca2+]o\begin{document}$[Ca^{2+}]_{o}$\end{document} dependent scaling of release probability UHill\begin{document}$U_{Hill}$\end{document}: mM, the release probability USE\begin{document}$U_{SE}$\end{document}, the average number of vesicles in the release-ready pool NRRP\begin{document}$N_{RRP}$\end{document}, and the NMDA/AMPA ratio g^ratio\begin{document}$\hat{g}_{ratio}$\end{document} are dimensionless. [file elife-99693-supp2.pdf]

Synaptic parameters of excitatory pathways. Average class parameters are marked in bold and are used predictively (in lack of reference *in vitro* data) for the remaining pathways belonging to the same class. Physical dimensions are as follows: peak conductance $\hat{g}$: nS, depression and facilitation time constants D, F, and the EPSC τ_{decay}: ms, the Hill coefficient of the nonlinear $[Ca^{2+}]_o$ dependent scaling of release probability U_{Hill}: mM, the release probability U_{SE}, the average number of vesicles in the release-ready pool N_{RRP}, and the NMDA/AMPA ratio $\hat{g}_{ratio}$ are dimensionless.

Pre	Post	$\hat{g}$	U_{SE}	D	F	N_{RRP}	τ_{decay}	$\hat{g}_{ratio}$	U_{Hill}
				**PC to Sst+IN (E1)**					
PC	MC	0.2±0.1	0.09±0.12	138±211	670±830	1.5	1.74±0.18	0.8	2.79
**PC**	**PT**	**0.6±0.5**	**0.02±0.00**	**194±18**	**507±37**	**4.5**	**1.74±0.18**	**0.8**	**1.09**
**PC**	**DT**	**0.2±0.1**	**0.02±0.00**	**194±18**	**507±37**	**1.5**	**1.74±0.18**	**0.8**	**2.79**
				**PC to PC (E2)**					
L23_PC	L23_PC	1.0±0.5	0.46±0.26	671±17	17±5	2.6	1.74±0.18	0.7	2.79
L4_PC	L4_PC	0.6±0.3	0.86±0.09	671±17	17±5	1.0	1.74±0.18	0.9	2.79
L4_SSC	L23_PC	0.2±0.1	0.79±0.04	671±17	17±5	1.8	1.74±0.18	0.5	2.79
L5_STPC	L5_STPC	0.9±0.3	0.39±0.03	690±90	44±21	1.0	1.74±0.18	0.7	2.79
L5_TTPC	L5_TTPC	1.9±1.0	0.38±0.10	365±100	25±45	2.8	1.74±0.18	0.7	2.79
L23_PC	L5_TTPC	0.5±0.2	0.50±0.02	671±17	17±5	1.5	1.74±0.18	0.7	2.79
L4_SSC	L5_STPC	0.6±0.3	0.50±0.02	671±17	17±5	1.2	1.74±0.18	0.7	2.79
L4_SSC	L6_PC	0.4±0.2	0.50±0.02	671±17	17±5	1.0	1.74±0.18	0.7	2.79
L6_TPC:A	L6_TPC:A	1.0±0.5	0.37±0.11	280±90	90±80	1.0	1.74±0.18	0.7	2.79
L6_TPC:C	L6_TPC:C	0.5±0.2	0.23±0.06	420±340	200±130	1.0	1.74±0.18	0.7	2.79
L6_IPC	L6_IPC	0.9±0.3	0.23±0.06	420±340	200±130	1.0	1.74±0.18	0.7	2.79
**L6_PC to same L6_PC**		**0.8±0.2**	**0.23±0.06**	**420±340**	**200±130**	**1.0**	**1.74±0.18**	**0.7**	**2.79**
L6_TPC:A	L6_TPC:C	1.2±0.5	0.23±0.06	420±340	200±130	1.0	1.74±0.18	0.7	2.79
L6_TPC:A	L6_BPC	0.3±0.1	0.23±0.06	420±340	200±130	1.0	1.74±0.18	0.7	2.79
L6_TPC:C	L6_IPC	0.2±0.1	0.23±0.06	420±340	200±130	1.0	1.74±0.18	0.7	2.79
L6_IPC	L6_BPC	0.4±0.1	0.23±0.06	420±340	200±130	1.0	1.74±0.18	0.7	2.79
L6_BPC	L6_TPC:A	0.2±0.1	0.23±0.06	420±340	200±130	1.0	1.74±0.18	0.7	2.79
**L6_PC to diff. L6_PC**		**0.5±0.4**	**0.23±0.06**	**420±340**	**200±130**	**1.0**	**1.74±0.18**	**0.7**	**2.79**
**PC**	**PC**	**0.7±0.4**	**0.50±0.02**	**671±17**	**17±5**	**1.5**	**1.74±0.18**	**0.7**	**2.79**
				**PC to Sst-IN (E2)**					
PC	NBC	0.6±0.4	0.72±0.12	227±70	13±24	4.5	1.74±0.18	0.8	1.09
**PC**	**PT**	**0.6±0.5**	**0.50±0.02**	**671±17**	**17±5**	**4.5**	**1.74±0.18**	**0.8**	**1.09**
PC	L1_GABAB-	0.3±0.1	0.50±0.02	671±17	17±5	1.5	1.74±0.18	0.8	1.94
L6_TPC:A	L6_BC	0.4±0.1	0.58±0.13	240±80	70±90	1.5	1.74±0.18	0.8	1.09
L6_TPC:C	L6_BC	0.4±0.1	0.36±0.21	380±310	280±340	1.5	1.74±0.18	0.8	1.09
L6_IPC	L6_BC	0.3±0.1	0.51±0.20	440±300	100±50	1.5	1.74±0.18	0.8	1.09
**L6_PC**	**L6_BC**	**0.4±0.1**	**0.47±0.21**	**370±290**	**155±215**	**1.5**	**1.74±0.18**	**0.8**	**1.09**
**PC**	**IN**	**0.4±0.1**	**0.50±0.02**	**671±17**	**17±5**	**1.5**	**1.74±0.18**	**0.8**	**1.94**

Proximal Targeting inhibitory mtypes: N/L/SBC, CHC. N/LBC etypes: cACint, cIR, bAC, bIR, cNAC have E1, while the rest of etypes; and SBC and CHC mtypes have E2 short-term dynamics.
Distal Targeting inhibitory mtypes: BP, DBC, BTC. L1_GABAB- comprise all non NGC mtypes in L1
