# Supplementary material for: Modeling and simulation of neocortical micro- and mesocircuitry (Part II, Physiology and experimentation)
Source: eLife. 2026 Jan 20;13:RP99693. doi: 10.7554/eLife.99693 (PMC12818871; doi:10.7554/eLife.99693)
Supplement: Supplementary file 3. — Average class parameters are marked in bold and are used predictively (in lack of reference in vitro data) for the remaining pathways belonging to the same class. Physical dimensions are as follows: peak conductance g^\begin{document}$\hat{g}$\end{document}: nS, depression and facilitation time constants D\begin{document}$D$\end{document}, F\begin{document}$F$\end{document}, and the IPSC τdecay\begin{document}$\tau_{decay}$\end{document}: ms, the Hill coefficient of the nonlinear [Ca2+]o\begin{document}$[Ca^{2+}]_{o}$\end{document} dependent scaling of release probability UHill\begin{document}$U_{Hill}$\end{document}: mM, the release probability USE\begin{document}$U_{SE}$\end{document}, the average number of vesicles in the release-ready pool NRRP\begin{document}$N_{RRP}$\end{document}, and the GABAB/GABAA\begin{document}$GABA_{B}/GABA_{A}$\end{document} ratio g^ratio\begin{document}$\hat{g}_{ratio}$\end{document} are dimensionless. [file elife-99693-supp3.pdf]

Synaptic parameters of inhibitory pathways. Average class parameters are marked in bold and are used predictively (in lack of reference *in vitro* data) for the remaining pathways belonging to the same class. Physical dimensions are as follows: peak conductance $\hat{g}$: nS, depression and facilitation time constants D, F, and the IPSC τ_{decay}: ms, the Hill coefficient of the nonlinear $[Ca^{2+}]_o$ dependent scaling of release probability U_{Hill}: mM, the release probability U_{SE}, the average number of vesicles in the release-ready pool N_{RRP}, and the $GABA_B/GABA_A$ ratio $\hat{g}_{ratio}$ are dimensionless.

Pre	Post	$\hat{g}$	U_{SE}	D	F	N_{RRP}	τ_{decay}	$\hat{g}_{ratio}$	U_{Hill}
				**IN to PC (I1)**					
L6_BC	L6_BC	2.3±0.5	0.16±0.10	45±21	376±253	1.0	10.40±6.20	0.0	1.94
SBC (cACint)	PC	1.9±1.0	0.16±0.10	45±21	376±253	3.3	10.40±6.20	0.0	1.94
				**IN to PC and IN (I2)**					
MC	PC	3.0±1.5	0.30±0.08	1250±520	2±4	1.0	8.30±2.20	0.0	1.94
**DT**	**PC**	**3.0±1.5**	**0.25±0.13**	**706±405**	**21±9**	**1.0**	**8.30±2.20**	**0.0**	**1.94**
NBC	PC	1.9±1.0	0.14±0.05	875±285	22±5	3.3	8.30±2.70	0.0	1.94
NGC	PC	0.2±0.1	0.25±0.13	706±405	21±9	1.0	36.50±1.30	0.8	1.94
L1_GABAB-	PC	0.3±0.1	0.25±0.13	706±405	21±9	1.0	8.30±2.20	0.0	1.94
SBC (dNAC)	PC	1.9±1.0	0.25±0.13	706±405	21±9	3.3	8.30±2.20	0.0	1.94
**IN**	*****	**2.3±0.5**	**0.25±0.13**	**706±405**	**21±9**	**1.0**	**8.30±2.20**	**0.0**	**1.94**
				**IN to PC (I3)**					
L6_BC	L6_PC	1.9±1.0	0.44±0.25	195±190	200±320	1.0	10.40±6.20	0.0	1.94
**PT**	**PC**	**1.9±1.0**	**0.32±0.14**	**144±80**	**62±31**	**3.3**	**6.40±1.70**	**0.0**	**1.94**

Proximal Targeting inhibitory mtypes: N/L/SBC, CHC. N/LBC etypes: cNAC, dSTUT, cSTUT, bSTUT have I3, while the rest of etypes; and SBC (except: cACint and dNAC) and CHC mtypes have I2 short-term dynamics. Distal Targeting inhibitory mtypes: BP, DBC, BTC. L1_GABAB- comprise all non NGC mtypes in L1
