# Supplementary material for: Modeling and simulation of neocortical micro- and mesocircuitry (Part II, Physiology and experimentation)
Source: eLife. 2026 Jan 20;13:RP99693. doi: 10.7554/eLife.99693 (PMC12818871; doi:10.7554/eLife.99693)
Supplement: Supplementary file 4. — Values taken from the internal connectivity (Supplementary file 2) are marked in bold. Physical dimensions are as follows: peak conductance g^\begin{document}$\hat{g}$\end{document}: nS, depression and facilitation time constants D\begin{document}$D$\end{document}, F\begin{document}$F$\end{document}, and the EPSC τdecay\begin{document}$\tau_{decay}$\end{document}: ms, the Hill coefficient of the nonlinear [Ca2+]o\begin{document}$[Ca^{2+}]_{o}$\end{document} dependent scaling of release probability UHill\begin{document}$U_{Hill}$\end{document}: mM, the release probability USE\begin{document}$U_{SE}$\end{document}, the average number of vesicles in the release-ready pool NRRP\begin{document}$N_{RRP}$\end{document}, and the NMDA/AMPA ratio g^ratio\begin{document}$\hat{g}_{ratio}$\end{document} are dimensionless. [file elife-99693-supp4.pdf]

**Synaptic parameters of thalamocortical pathways.** Values taken from the internal connectivity (Table **??**) are marked in bold. Physical dimensions are the same as in Table **??**.

| **Pre** | **Post** | $\hat{g}$ | $U_{SE}$ | $D$ | $F$ | $N_{RRP}$ | $\tau_{decay}$ | $\hat{g}_{ratio}$ | $U_{Hill}$ |
|---|---|---|---|---|---|---|---|---|---|
| | | | | **VPM, POm to Sst+IN (E1)** | | | | | |
| * | Sst+ | **0.2±0.1** | **0.09±0.12** | **138±211** | **670±830** | **1.5** | **1.74±0.18** | **0.8** | **2.79** |
| | | | | **VPM, POm to PC (E2)** | | | | | |
| VPM | L23_PC | 1.7±0.6 | 0.75±0.1 | **671±17** | **17±5** | **1.5** | **1.74±0.18** | **0.7** | **2.79** |
| VPM | L4_PC | 1.1±0.4 | 0.75±0.1 | **671±17** | **17±5** | **1.5** | **1.74±0.18** | **0.7** | **2.79** |
| VPM | L56_PC | 2.4±0.9 | 0.75±0.1 | **671±17** | **17±5** | **1.5** | **1.74±0.18** | **0.7** | **2.79** |
| POm | PC | 1.7±0.6 | 0.75±0.1 | **671±17** | **17±5** | **1.5** | **1.74±0.18** | **0.7** | **2.79** |
| | | | | **VPM, POm to PV+IN (E2)** | | | | | |
| VPM | L4_PV+ | 1.4±0.4 | **0.72±0.12** | **227±70** | **13±24** | **4.5** | **1.74±0.18** | **0.8** | **1.09** |
| VPM | L6_PV+ | 3.1±1.0 | **0.72±0.12** | **227±70** | **13±24** | **4.5** | **1.74±0.18** | **0.8** | **1.09** |
| VPM | L235_PV+ | 2.2±0.4 | **0.72±0.12** | **227±70** | **13±24** | **4.5** | **1.74±0.18** | **0.8** | **1.09** |
| POm | PV+ | 2.2±0.4 | **0.72±0.12** | **227±70** | **13±24** | **4.5** | **1.74±0.18** | **0.8** | **1.09** |
| | | | | **VPM, POm to 5HT3aR+IN (E2)** | | | | | |
| * | 5HT3aR+ | **0.4±0.1** | **0.50±0.02** | 671±17 | 17±5 | 1.5 | **1.74±0.18** | **0.8** | **1.94** |

Sst+ inhibitory mtypes: MC and BP, DBC, BTC (cACint etype only). PV+ inhibitory mtypes: N/LBC, CHC. 5HT3aR+ mtypes: the rest of the mtypes not listed above (e.g. NGC, SBC, and everything in L1).
