# Supplementary material for: Modeling and simulation of neocortical micro- and mesocircuitry (Part II, Physiology and experimentation)
Source: eLife. 2026 Jan 20;13:RP99693. doi: 10.7554/eLife.99693 (PMC12818871; doi:10.7554/eLife.99693)
Supplement: Supplementary file 5. — See Figure 3B1. [file elife-99693-supp5.pdf]

Validation of PSP amplitudes. See Figure 2B1.

**Pre**	**Post**	*in vitro* (mV)	*in silico* (mV)	Reference
L23_PC	L23_PC	1.00±0.70	0.99±0.67	***Feldmeyer et al.*** (***2006***)
L23_PC	L5_TTPC	0.30±0.30	0.30±0.24	***Reyes and Sakmann*** (***1999***)
L4_EXC	L4_EXC	1.59±1.51	1.62±1.31	***Feldmeyer et al.*** (***1999***)
L4_SSC	L23_PC	0.70±0.60	0.66±0.34	***Feldmeyer et al.*** (***2002***)
L4_SSC	L5_STPC	0.60±0.40	0.59±0.33	***Feldmeyer et al.*** (***2005***)
L4_SSC	L6_PC	0.29±0.16	0.30±0.30	***Qi and Feldmeyer*** (***2016***)
L5_TTPC	L5_TTPC	1.30±1.10	1.24±0.73	***Markram et al.*** (***1997***)
L5_STPC	L5_STPC	0.80±0.20	0.75±0.40	***Le Bé et al.*** (***2007***)
L6_BPC	L6_TPC:A	0.21±0.00	0.22±0.15	***Berger*** (***2009***)
L6_IPC	L6_BPC	0.42±0.18	0.42±0.21	***Berger*** (***2009***)
L6_IPC	L6_IPC	1.05±0.31	1.09±0.81	***Berger*** (***2009***)
L6_TPC:C	L6_IPC	0.18±0.00	0.19±0.13	***Berger*** (***2009***)
L6_TPC:C	L6_TPC:C	0.43±0.22	0.43±0.29	***Berger*** (***2009***)
L6_TPC:A	L6_BPC	0.32±0.27	0.31±0.17	***Berger*** (***2009***)
L6_TPC:A	L6_TPC:C	1.19±0.15	1.10±0.63	***Berger*** (***2009***)
L6_TPC:A	L6_TPC:A	1.51±0.98	1.45±1.11	***Berger*** (***2009***)
L23_PC	L1_GABAB-	1.10±0.30	1.09±0.83	***Wozny and Williams*** (***2011***)
L4_EXC	L4_FS	2.20±2.20	2.17±2.46	***Beierlein et al.*** (***2003***)
L5_TTPC	L5_MC	0.28±0.30	0.28±0.33	***Silberberg and Markram*** (***2007***)
L6_IPC	L6_BC	1.59±1.60	1.56±1.48	***Berger*** (***2009***)
L6_TPC:A	L6_BC	2.20±3.28	2.02±1.44	***Berger*** (***2009***)
L6_TPC:C	L6_BC	1.29±1.65	1.28±0.84	***Berger*** (***2009***)
L6_PC	L6_MC	0.20±0.12	0.15±0.14	***Berger*** (***2009***)
L1_NGC	L23_PC	0.58±0.10	0.54±0.41	***Wozny and Williams*** (***2011***)
L1_GABAB-	L23_PC	0.27±0.04	0.26±0.13	***Wozny and Williams*** (***2011***)
L4_FS	L4_EXC	1.10±0.80	1.14±0.81	***Beierlein et al.*** (***2003***)
L5_MC	L5_TTPC	0.50±0.40	0.47±0.25	***Silberberg and Markram*** (***2007***)
VPM	L4_EXC	2.40±2.00	2.51±2.10	***Beierlein et al.*** (***2003***)
VPM	L4_FS	4.10±3.20	4.09±2.52	***Beierlein et al.*** (***2003***)
VPM	L6_EXC	1.20±0.80	1.28±1.96	***Beierlein and Connors*** (***2002***)
VPM	L6_FS	3.90±3.50	3.11±2.57	***Beierlein and Connors*** (***2002***)

Thick-tufted mtypes: L5_TPC:A, L5_TPC:B. Slender-tufted mtypes: L5_TPC:C, L5_UPC.

References

Beierlein M, Connors BW. Short-term dynamics of thalamocortical and intracortical synapses onto layer 6 neurons in neocortex. Journal of Neurophysiology. 2002; 88(4):1924–1932. doi: 10.1152/jn.2002.88.4.1924.

Beierlein M, Gibson JR, Connors BW. Two Dynamically Distinct Inhibitory Networks in Layer 4 of the Neocortex. Journal of Neurophysiology. 2003; 90(5):2987–3000. doi: 10.1152/jn.00283.2003.

Berger T. Properties of Neocortical Microcircuits. PhD thesis, Ecole Polytechnique Fédérale de Lausanne; 2009.

Feldmeyer D, Egger V, Lübke J, Sakmann B. Reliable synaptic connections between pairs of excitatory layer 4 neurones within a single 'barrel' of developing rat somatosensory cortex. Journal of Physiology. 1999; 521(1):169–190. doi: 10.1111/j.1469-7793.1999.00169.x.

Feldmeyer D, Lübke J, Sakmann B. Efficacy and connectivity of intracolumnar pairs of layer 2/3 pyramidal cells in the barrel cortex of juvenile rats. Journal of Physiology. 2006; 575(2):583–602. doi: 10.1113/jphysiol.2006.105106.

Feldmeyer D, Lübke J, Silver RA, Sakmann B. Synaptic connections between layer 4 spiny neurone-layer 2/3 pyramidal cell pairs in juvenile rat barrel cortex: Physiology and anatomy of interlaminar signalling within a cortical column. Journal of Physiology. 2002; 538(3):803–822. doi: 10.1113/jphysiol.2001.012959.

Feldmeyer D, Roth A, Sakmann B. Monosynaptic connections between pairs of spiny stellate cells in layer 4 and pyramidal cells in layer 5A indicate that lemniscal and paralemniscal afferent pathways converge in the infragranular somatosensory cortex. Journal of Neuroscience. 2005; 25(13):3423–3431. doi: 10.1523/JNEUROSCI.5227-04.2005.

Le Bé JV, Silberberg G, Wang Y, Markram H. Morphological, electrophysiological, and synaptic properties of corticocallosal pyramidal cells in the neonatal rat neocortex. Cerebral Cortex. 2007; 17(9):2204–2213. doi: 10.1093/cercor/bhl127.

Markram H, Lübke J, Frotscher M, Roth A, Sakmann B. Physiology and anatomy of synaptic connections between thick tufted pyramidal neurones in the developing rat neocortex. Journal of Physiology. 1997; 500(2):409–440. doi: 10.1113/jphysiol.1997.sp022031.

Qi G, Feldmeyer D. Dendritic Target Region-Specific Formation of Synapses between Excitatory Layer 4 Neurons and Layer 6 Pyramidal Cells. Cerebral Cortex. 2016; 26(4):1569–1579. doi: 10.1093/cercor/bhu334.

Reyes A, Sakmann B. Developmental switch in the short-term modification of unitary EPSPs evoked in layer 2/3 and layer 5 pyramidal neurons of rat neocortex. Journal of Neuroscience. 1999; 19(10):3827–3835. doi: 10.1523/jneurosci.19-10-03827.1999.

Silberberg G, Markram H. Disynaptic Inhibition between Neocortical Pyramidal Cells Mediated by Martinotti Cells. Neuron. 2007; 53(5):735–746. doi: 10.1016/j.neuron.2007.02.012.

Wozny C, Williams SR. Specificity of synaptic connectivity between layer 1 inhibitory interneurons and layer 2/3 pyramidal neurons in the rat neocortex. Cerebral Cortex. 2011; 21(8):1818–1826. doi: 10.1093/cercor/bhq257.
