# Supplementary material for: Modeling and simulation of neocortical micro- and mesocircuitry (Part II, Physiology and experimentation)
Source: eLife. 2026 Jan 20;13:RP99693. doi: 10.7554/eLife.99693 (PMC12818871; doi:10.7554/eLife.99693)
Supplement: Supplementary file 6. — See Figure 3B2. [file elife-99693-supp6.pdf]

Validation of first PSP amplitudes' CVs. See Figure 2B2.

**Pre**	**Post**	*in vitro* (mV)	*in silico* (mV)	Reference
L4_EXC	L4_EXC	0.37±0.16	0.25±0.06	***Feldmeyer et al.*** (***1999***)
L4_EXC	L4_FS	0.27±0.13	0.38±0.25	***Beierlein et al.*** (***2003***)
L4_FS	L4_EXC	0.25±0.11	0.28±0.09	***Beierlein et al.*** (***2003***)
L4_SS	L5_STPC	0.33±0.20	0.43±0.06	***Feldmeyer et al.*** (***2005***)
L4_SS	L6_PC	0.50±0.11	0.51±0.06	***Qi and Feldmeyer*** (***2016***)
L4_SS	L23_PC	0.27±0.13	0.32±0.07	***Feldmeyer et al.*** (***2002***)
L5_TTPC	L5_TTPC	0.31±0.14	0.39±0.09	***Barros-Zulaica et al.*** (***2019***)
L5_STPC	L5_STPC	0.58±0.24	0.51±0.06	***Le Bé et al.*** (***2007***)
L23_PC	L23_PC	0.33±0.18	0.43±0.15	***Feldmeyer et al.*** (***2006***)
L234_PC	L234_NBC	0.32±0.08	0.21±0.08	***Wang et al.*** (***2002***)

Thick-tufted mtypes: L5_TPC:A, L5_TPC:B. Slender-tufted mtypes: L5_TPC:C, L5_UPC.

References

Barros-Zulaica N, Rahmon J, Chindemi G, Perin R, Markram H, Muller E, Ramaswamy S. Estimating the Readily-Releasable Vesicle Pool Size at Synaptic Connections in the Neocortex. Frontiers in Synaptic Neuroscience. 2019; 11(29). doi: 10.3389/fnsyn.2019.00029.

Beierlein M, Gibson JR, Connors BW. Two Dynamically Distinct Inhibitory Networks in Layer 4 of the Neocortex. Journal of Neurophysiology. 2003; 90(5):2987–3000. doi: 10.1152/jn.00283.2003.

Feldmeyer D, Egger V, Lübke J, Sakmann B. Reliable synaptic connections between pairs of excitatory layer 4 neurones within a single 'barrel' of developing rat somatosensory cortex. Journal of Physiology. 1999; 521(1):169–190. doi: 10.1111/j.1469-7793.1999.00169.x.

Feldmeyer D, Lübke J, Sakmann B. Efficacy and connectivity of intracolumnar pairs of layer 2/3 pyramidal cells in the barrel cortex of juvenile rats. Journal of Physiology. 2006; 575(2):583–602. doi: 10.1113/jphysiol.2006.105106.

Feldmeyer D, Lübke J, Silver RA, Sakmann B. Synaptic connections between layer 4 spiny neurone-layer 2/3 pyramidal cell pairs in juvenile rat barrel cortex: Physiology and anatomy of interlaminar signalling within a cortical column. Journal of Physiology. 2002; 538(3):803–822. doi: 10.1113/jphysiol.2001.012959.

Feldmeyer D, Roth A, Sakmann B. Monosynaptic connections between pairs of spiny stellate cells in layer 4 and pyramidal cells in layer 5A indicate that lemniscal and paralemniscal afferent pathways converge in the infragranular somatosensory cortex. Journal of Neuroscience. 2005; 25(13):3423–3431. doi: 10.1523/JNEUROSCI.5227-04.2005.

Le Bé JV, Silberberg G, Wang Y, Markram H. Morphological, electrophysiological, and synaptic properties of corticocallosal pyramidal cells in the neonatal rat neocortex. Cerebral Cortex. 2007; 17(9):2204–2213. doi: 10.1093/cercor/bhl127.

Qi G, Feldmeyer D. Dendritic Target Region-Specific Formation of Synapses between Excitatory Layer 4 Neurons and Layer 6 Pyramidal Cells. Cerebral Cortex. 2016; 26(4):1569–1579. doi: 10.1093/cercor/bhu334.

Wang Y, Gupta A, Toledo-Rodriguez M, Wu CZ, Markram H. Anatomical, physiological, molecular and circuit properties of nest basket cells in the developing somatosensory cortex. Cerebral cortex. 2002; 12(4):395–410. doi: 10.1093/cercor/12.4.395.
