# Supplementary material for: Modeling and simulation of neocortical micro- and mesocircuitry (Part II, Physiology and experimentation)
Source: eLife. 2026 Jan 20;13:RP99693. doi: 10.7554/eLife.99693 (PMC12818871; doi:10.7554/eLife.99693)
Supplement: Supplementary file 7. — See Figure 3E2. [file elife-99693-supp7.pdf]

Table 1. Validation of mPSC frequency. See Figure 2E2.

**Pre**	**Post**	*in vitro* (Hz)	*in silico* (Hz)	Reference
E	L23_PC	8.20±2.90	9.36±4.38	***Brasier and Feldman*** (***2008***)
E	L4_PC	11.90±2.40	15.64±7.84	***Brasier and Feldman*** (***2008***)
E	L6_CC	2.80±0.80	3.87±2.14	***Yang et al.*** (***2020***)
E	L6_CT	0.95±0.36	1.41±0.74	***Yang et al.*** (***2020***)
I	L5_PC	21.10±4.80	16.06±6.74	***Ling and Benardo*** (***1999***)

Cortico-cortical mtypes: L6_UPC, L6_IPC, L6_HPC.
Cortico-thalamic mtypes: L6_TPC:A, L6_TPC:C.

References

Brasier DJ, Feldman DE. Synapse-specific expression of functional presynaptic NMDA receptors in rat somatosensory cortex. Journal of Neuroscience. 2008; 28(9):2199–2211. doi: 10.1523/JNEUROSCI.3915-07.2008.

Ling DSF, Benardo LS. Restrictions on inhibitory circuits contribute to limited recruitment of fast inhibition in rat neocortical pyramidal cells. Journal of Neurophysiology. 1999; 82(4):1793–1807. doi: 10.1152/jn.1999.82.4.1793.

Yang D, Günter R, Qi G, Radnikow G, Feldmeyer D. Muscarinic and Nicotinic Modulation of Neocortical Layer 6A Synaptic Microcircuits Is Cooperative and Cell-Specific. Cerebral Cortex. 2020; 30(6):3528–3542. doi: 10.1093/cercor/bhz324.
