# Supplementary material for: Modeling and simulation of neocortical micro- and mesocircuitry (Part II, Physiology and experimentation)
Source: eLife. 2026 Jan 20;13:RP99693. doi: 10.7554/eLife.99693 (PMC12818871; doi:10.7554/eLife.99693)
Supplement: Supplementary file 8. — Additionally, we assume that various input data generalizations between brain regions, organisms and animal ages do not affect validity. These are not listed again. [file elife-99693-supp8.pdf]

Assumptions. Additionally, we assume that various input data generalizations between brain regions, organisms and animal ages do not affect validity. They are not listed again.

Anatomy

Inherited

All assumptions about the model's anatomy are inherited from the companion paper (***Reimann et al., 2024***), where they are described in Section 5.2.

Neuron Physiology

Structuring

We assume that me-types are sufficient to capture the diversity of neuron dynamics. In particular, this includes a single firing type for pyramidal cells and no met-types. See discussion for further details.

Generalization

Optimization of ion channel densities to match somatic electrophysiological features, creates accurate dendritic activity. Whilst we validated several dendritic properties, the battery of tests could be increased. Moreover, multimodal fitting approaches can now be used to construct single-neuron electrical models with patch clamp and high-density microelectrode arrays (***Buccino et al., 2024***).

The model assumes that electrical models optimized for a given me-type can generalize to different m-types of the same e-type. Importantly, the quality of generalization is tested, with poor generalizations rejected. As optimization methods become more efficient and more data is available, such assumptions can be relaxed. Future work would benefit from single cell protocols applied to many different met-types.

Modeling

11 generic ion channels are sufficient to capture accurate neuronal dynamics. We discuss how larger sets of ion channel models could be used in future.

The model assumes that ion channel densities are constant for each neurite type (soma, axon initial segment, apical and basal dendrites), except for HCN channels (which increase exponentially with distance from the soma in apical and basal dendrites) and Na^{2+} channels (which decrease exponentially with distance from the soma in apical dendrites). More data on the spatial distribution of ion channels for different neuron types would be very useful to constraining future optimizations.

Data

In vitro recordings of single cell protocols are sufficiently informative of *in vivo* dynamics.

Synaptic Physiology

Structuring

The model assumes that synaptic physiology is specific to m-types, rather than me-types or even more specific classes.

Generalization

The model assumes that for the fitting of synaptic parameters, PSP data can be generalized to pathways with missing data. New work in mouse presents techniques for recording PSPs at larger scale.

Modeling

Modeling the NMDA Mg^{2+} block is sufficient to capture the voltage-dependent nature of NMDA currents.

Missing input compensation

Modeling

Input compensation at the soma can capture the effect of missing input. This is further considered in the Discussion. Comparing to glutamate uncaging studies would test the dendritic processing characteristics of neurons under different missing input compensation schemes.

Inputs from other brain regions are independent between neurons. In the future, it is possible that temporal synchronization of different brain regions recorded in openly available brain wide electrophysiology studies could be combined with innervation patterns from other brain regions to better constrain the synchrony of inputs.

Structuring

Missing input is specific for layer-wise E/I populations. Input compensation could be made more specific, e.g., for inhibitory subpopulations.

Data

Spike sorting bias is constant across neuron populations. Our latest work ***Laquitaine et al.*** (***2024***) predicts how biases could be different between subpopulations. These predictions could be incorporated in future work.

Thalamic stimulus

Modeling

We assume that thalamic neurons activated in a single whisker deflection are randomly distributed in a barreloid of the VPM corresponding to a cortical barrel, and that individual neuron spikes are drawn from a population PSTH. To our knowledge there is little data about the spatial distribution of thalamic fibers activated during a specific whisker stimulus. Combined intracellular recording of thalamacortical projection neurons stimulated during a single whisker deflection and an anatomical reconstruction of the stimulated fibers would improve the pattern of synaptic activations innervating the cortex and allow more precise modeling of single neuron and network integration. This goal is now much closer, given that the temporal population response of our model is now accurate on the millisecond timescale.

Comparison of evoked activity to spike sorted recordings

Data

We additionally assume that similarity with spike sorted PSTHs is sufficient for validation of stimulus evoked activity. As discussed through-out the paper, such recordings contain bias, and also do not compare against the internal dynamics of single neurons. We believe this paper sets the stage for meaningful comparisons to a large quantity of existing analyses of intracellular recordings in the barrel cortex. Calcium imaging also offers a useful measure of sparsity in L23 in both spontaneous and evoked settings, and could simultaneously be compared alongside intracellular responses to active whisker touch stimuli (***O'Connor et al., 2010***). The mouse barrel cortex would offer an excellent model for reconciling a broad range of previous findings at these levels. Our model's evoked response can now also be compared directly to local field potential data as in (***Rimehaug et al., 2023***).

References

Buccino AP, Damart T, Bartram J, Mandge D, Xue X, Zbili M, Gänswein T, Jaquier A, Emmenegger V, Markram H, et al. A Multimodal Fitting Approach to Construct Single-Neuron Models With Patch Clamp and High-Density Microelectrode Arrays. Neural Computation. 2024; 36(7):1286–1331.

Laquitaine S, Imbeni M, Tharayil J, Isbister JB, Reimann MW. Spike sorting biases and information loss in a detailed cortical model. bioRxiv. 2024; https://www.biorxiv.org/content/early/2024/12/07/2024.12.04.626805, doi: 10.1101/2024.12.04.626805.

O'Connor DH, Peron SP, Huber D, Svoboda K. Neural activity in barrel cortex underlying vibrissa-based object localization in mice. Neuron. 2010; 67(6):1048–1061.

Reimann MW, Bolaños-Puchet S, Courcol JD, Egas Santander D, Arnaudon A, Coste B, Delalondre F, Delemontex T, Devresse A, Dictus H, Dietz A, Ecker A, Favreau C, Ficarelli G, Gevaert M, Herttuainen J, Isbister JB, Kanari L, Keller D, King J, et al. Modeling and Simulation of Neocortical Micro- and Mesocircuitry. Part I: Anatomy. eLife. 2024; doi: 10.7554/elife.99688.1.

Rimehaug AE, Stasik AJ, Hagen E, Billeh YN, Siegle JH, Dai K, Olsen SR, Koch C, Einevoll GT, Arkhipov A. Uncovering circuit mechanisms of current sinks and sources with biophysical simulations of primary visual cortex. elife. 2023; 12:e87169.
